# Chromosomal variation in Argentine populations of *Akodon montensis* Thomas, 1913 (Rodentia, Cricetidae, Sigmodontinae)

**DOI:** 10.3897/CompCytogen.v10i1.6420

**Published:** 2016-02-02

**Authors:** Matías Maximiliano Malleret, Carolina Alicia Labaroni, Gabriela Verónica García, Juan Martín Ferro, Dardo Andrea Martí, Cecilia Lanzone

**Affiliations:** 1 Laboratorio de Genética Evolutiva (LGE) FCEQyN, IBS (CONICET-UNaM), Nodo Posadas, Félix de Azara 1552, CP3300, Posadas, Misiones, Argentina; 2 IBS (CONICET-UNaM), Nodo Iguazú, Bertoni 85, CP3370, Puerto Iguazú, Misiones, Argentina

**Keywords:** Rodents, karyotype variability, chromosome banding, heterochromatin, Ag-NORs

## Abstract

The genus *Akodon* Meyen, 1833 is one of the most species-rich among sigmodontine rodents and has great chromosome variability. *Akodon montensis* has a relatively broad distribution in South America, and Argentine populations are located in the southernmost region of its range. Brazilian populations have important chromosomal variability, but cytogenetic data from Argentina are scarce. We performed a chromosome characterization of natural populations of *Akodon montensis* using conventional staining, C-banding, Ag-NORs and base-specific fluorochromes. A total of 31 specimens from five localities of Misiones Province, in Argentina, were analyzed. The 2n=24 chromosomes was the most frequently observed karyotype. However, five individuals presented 25 chromosomes due to a supernumerary B-chromosome; and one individual had 2n=26 due to one B plus a trisomy for chromosome 11. Additionally, two XY females and two variants of the X chromosomes were found. C-positive centromeric bands occurred in all chromosomes; additional C-bands were observed in some autosomes, the X, Y and B chromosomes. Ag-NORs were observed in five autosomes, and the B chromosome was frequently marked. Fluorochrome banding was similar among karyotypes of the analyzed populations. Comparisons of cytogenetic data among populations of Argentina and Brazil showed the presence of high intraspecific variability in *Akodon montensis* and some differences among regions.

## Introduction

The genus *Akodon* Meyen, 1983, with about 41 species, is considered one of the most species-rich group within the subfamily Sigmodontinae. Its species are widely distributed in South America and inhabit a variety of habitats, among subtropical and tropical moist forest as well as desert regions (Musser and Carleton 2005). From a taxonomic point of view, the genus includes morphologically very similar species, and cytogenetic data is valuable for identifying them, such as *Akodon cursor* (Winge, 1887) and *Akodon montensis* (Yonenaga-Yassuda et al. 1975; Barros et al. 2009).This genus has high karyotypic variability, with chromosome numbers varying from 2n=46 (FN=46) in *Akodon serrensis* Thomas, 1902 to 2n=10 (FN=14) in *Akodon* sp. (Barros et al. 2009). In different species, several chromosome variations were described, including pericentric inversions and Robertsonian translocations in autosomes, modifications of sex chromosomes and the presence of B chromosomes (Silva and Yonenaga-Yassuda 1998; Fernández-Donoso et al. 2001; Bianchi 2002).

*Akodon montensis* is an abundant species distributed in Argentina, Brazil and Paraguay, and has a great chromosomal variability (Kasahara and Yonenaga-Yassuda 1982; Musser and Carleton 2005). Previous cytogenetic analysis demonstrated that the standard chromosome complement of *Akodon montensis* is 2n=24 (FN=42), with both X and Y chromosomes acrocentric (Yonenaga-Yassuda et al. 1975; Kasahara and Yonenaga-Yassuda 1982; Liascovich and Reig 1989). However, for animals from Brazil, Kasahara and Yonenaga-Yassuda (1982) described a morphological variation for the X chromosome, which was present in both sexes. In populations of Brazil XY fertile females were detected using specific DNA probes from the Y chromosome (Fagundes et al. 2000). Additionally the presence of supernumerary or B chromosomes was reported for specimens from Brazil (Yonenaga-Yassuda et al. 1975; Kasahara and Yonenaga-Yassuda 1982; Di-Nizo et al. 2014). Cytogenetic data on natural populations of *Akodon montensis* in Argentina are scarce. Liascovich and Reig (1989) studied four specimens from the Provincial Park “Islas Malvinas”, in Misiones Province; all specimens had no variations in the standard complement.

In order to contribute to the knowledge of karyotypic variability in *Akodon montensis* we analyzed specimens from different localities of Misiones Province, Argentina, which is a part of the southernmost area of the range (Pardiñas and Teta 2006).

## Material and methods

**Figure 1.**
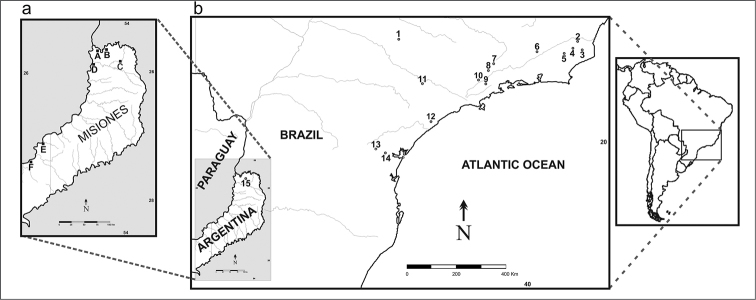
Map indicating **a** collection sites of *Akodon montensis* in the province of Misiones, Argentina analyzed in this work: **A** and **B** Iguazú **C** Parque Provincial Urugua-í **D** Puerto Esperanza **E** San Ignacio **F** Candelaria **b** different localities in Brazil and Argentina where *Akodon montensis* has been studied previously at cytogenetic level; Brazil: **1** Boracéia **2** Sumidouro **3** Nova Friburgo **4** Teresópolis **5** Petrópolis **6** S. J. do Barreiro **7** Taubaté **8** Caçapava **9** Salesópolis **10** Guararema **11** Itapetininga **12** Iguape **13** Quatro Barras **14** Tres barras; Argentina: **15** Misiones, Urugua-í.

A total of 31 specimens of *Akodon montensis* (18 females and 13 males) were collected from five localities of Misiones Province, Argentina (Fig. 1). Vouchers were deposited in the biological collection of the Instituto de Biología Subtropical (IBS-CONICET-UNaM). Chromosome preparations were obtained from bone marrow and testes (Ford and Hamerton 1956; Evans et al. 1964). Ten metaphase spreads were counted for each specimen, except in the individual with trisomy in which we counted 30. Conventional staining was performed with Giemsa (10%) to construct karyotypes. The distribution of constitutive heterochromatin (C-bands) was determined according to Sumner (1972) method. In order to identify chromosome homology and characterize sequences rich in AT and GC base pairs, the staining with the fluorochromes DAPI (4,6-diamidino-2-phenylindole) and CMA3 (Chromomicine A_3_) respectively, were conducted according to Schweizer´s method (1976, 1980). Ag-NORs staining was performed with the technique proposed by Howell and Black (1980) to detect active nucleolus organizer regions (NORs). In order to test if NORs carried by the B chromosome have any effect on the activation of autosomal NORs we made Student´s tests using INFOSTAT software.

## Results

**Figure 2.**
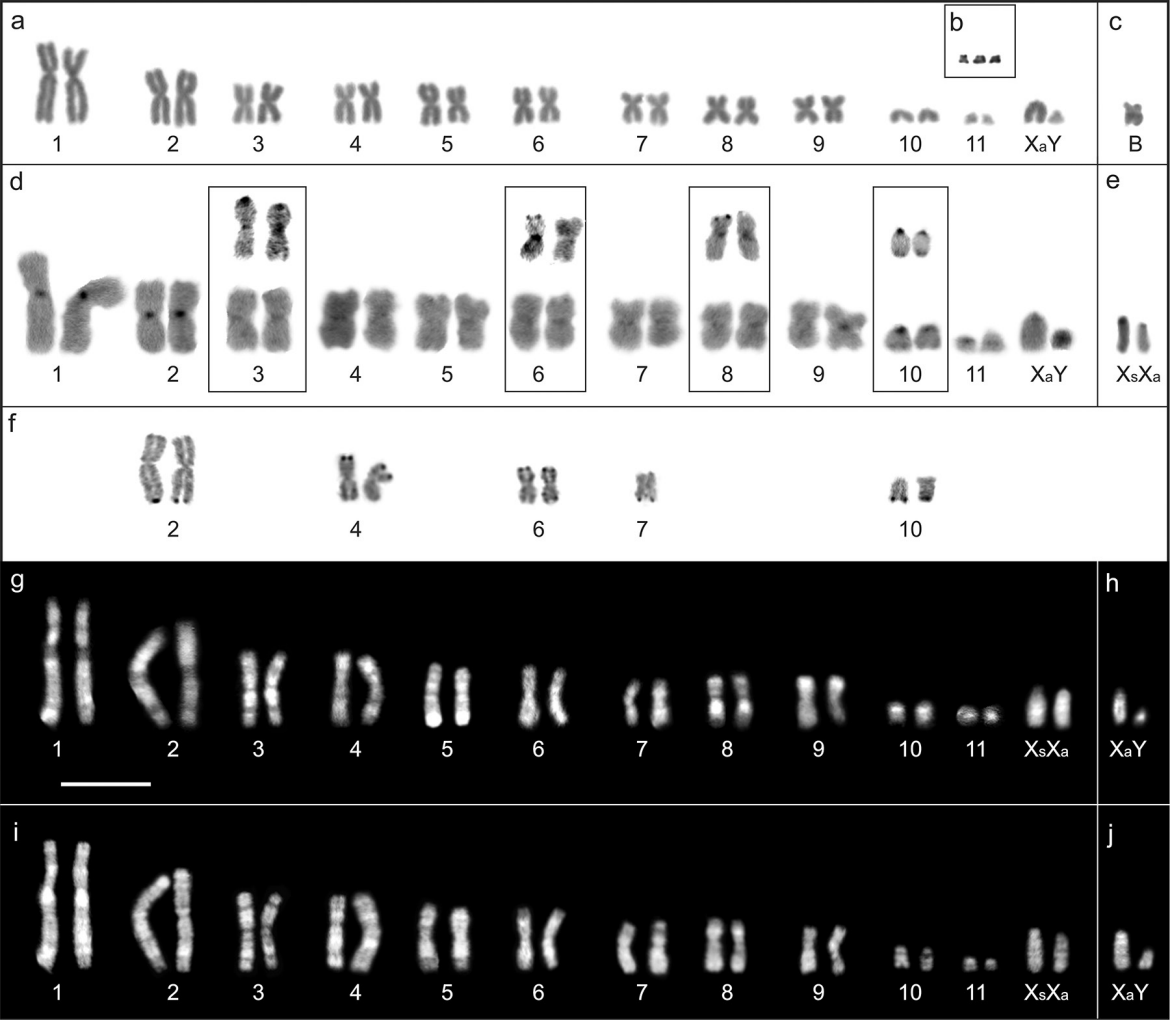
Mitotic chromosomes of *Akodon montensis*: **a** Giemsa stained karyotype of a male with 2n=24; FN=42 **b** the trisomy for pair 11 **c** Giemsa stained B chromosome **d** C-banded karyotype of a male, in the boxes pairs with telomeric C-bands are showed **e** C-band pattern of Xs-Xa sex chromosomes **f** Ag-NORs bearing chromosomes **g, i** karyotypes of a female with DAPI/ CMA_3_ fluorochrome staining respectively **h, j** DAPI/CMA_3_ fluorochrome pattern of sex chromosomes of a male. Bar = 10 µm.

All individuals of *Akodon montensis* had an autosome complement composed of nine pairs of large to medium size metacentric chromosomes, and two small-sized pairs, one acrocentric and one metacentric. The sex chromosome pair is XX/XY (Fig. 2).

Twenty-five individuals (fourteen females and eleven males) presented a karyotype with 2n=24 and FN=42 (Fig. 2a). Five specimens (four females and one male) had 25 chromosomes in all analyzed cells due to the presence of a small submetacentric B chromosome (Fig. 2c). The supernumerary chromosome was found in the five localities, representing 19.35% of the total sample. Only one male had 26 chromosomes in all analyzed cells (N=30) due to one B and to a trisomy for pair 11 (Fig. 2b, Table 1).

**Table 1.** Sampling localities of *Akodon montensis* analyzed in this work. Geographical coordinates, N=number of individuals indicating females (F) and males (M), 2n=chromosome number, sex chromosomes morphology for the X (Xa=acrocentric, Xs=subtelocentric, the number of individuals with each genotype are indicated in bracket), and frequency of B chromosome in each locality (F_B_).

Locality (Lat/Long)	N	2n		Sex Chromosome types	F_B_
		24	25 (24+B)		
Iguazú (25°42.08'S; 54°20.68'W)	10F	8	2	XaXa(7)	0.13
				XsXa (2)	
				XsY (1)**	
	6M	6	-	XaY (6)	
San Ignacio (27°16.88'S; 55°34.72'W)	2F	1	1	XsXa (1) XaXa (1)	0.25
	2M	2	-	XaY (2)	
Puerto Esperanza (25°59.23'S; 54°38.85'W)	4F	3	1	XsXa (3) XsXs (1)	0.25
Urugua-í (25°51.33'S; 54°10.02'W)	1F	1	-	XaXa (1)	0.20
	4M	3	1	XaY (4)	
Candelaria (27°22.79'S; 55°38.54'W)	1F	1	-	XaY (1)**	0.50
	1M	-	1*	XaY (1)	
**Total**	31	25	6	-	0.19

* an individual with one B and trisomy for pair 11

** the heterogametic females

**Figure 3.**
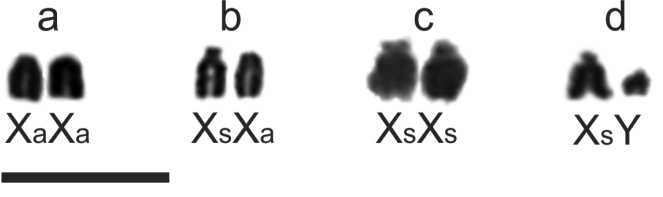
Variants of sex chromosomes in the females of *Akodon montensis* with Giemsa staining: **a** homozygous acrocentrics **b** acrocentric and subtelocentric **c** homozygous subtelocentrics **d** heterogametic sex chromosomes. Bar = 10 µm.

The Y chromosome was small acrocentric. The X was a medium-sized chromosome and showed two morphological variants: acrocentric (Xa) observed on both sexes, and subtelocentric (Xs) detected only for females (Figs 2, 3). From eighteen females, nine (56.25%) were homozygous for Xa (Fig. 3a), six (37.50%) were heterozygous (Fig. 3b), and one (6.25%) showed both Xs chromosomes (Fig. 3c). Additionally, two females apparently were heterogametic with XY chromosomes, the one from Iguazú had the Xs (Fig. 3d), and the other from Candelaria had Xa chromosome (Table 1).

**Figure 4.**
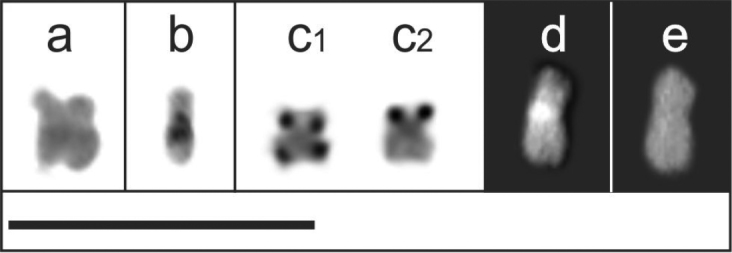
B chromosome of *Akodon montensis*: **a** Giemsa staining **b** C-banding, pericentromeric and interstitial C-bands **c** silver nitrate staining with Ag-NORs in both telomeric ends (**C1**) and single in the end of the short arm (**C2**) **d, e** DAPI/ CMA_3_ fluorochrome stained respectively. Bar = 10 µm.

Positive C-band (C+) were found in the pericentromeric region of pairs 1 to 11, and at the telomeres of pairs 3, 6, 8 and 10 (Fig. 2d). Acrocentric and subtelocentric variants of X chromosome had positive C-bands in the pericentromeric regions (Fig. 2d–e). Additionally, the subtelocentric X chromosome presented a large positive C-band, which covered its short arm (Fig. 2e). The Y chromosome was completely heterochromatic (Fig. 2d). The B chromosome showed two C+ bands, one was interstitial and the other pericentromeric (Fig. 4b).

Ag-NORs were evident in the distal position of pairs 2, 4, 6, 7 and 10 (Fig. 2f). However, the number of positive signals varied between two and seven in different cells (See Suppl. material 1). Pair 10 was active in most (92/100) analyzed cells. Additionally, the B chromosome frequently was stained (28/36 cells) in one or both telomeric ends (Fig. 4c). The total number of positive Ag-NORs was different between cells with B (four specimens, 36 cells, mean 5.639, SD=1.76) and without the B chromosome (ten specimens, 64 cells, mean 4.328, SD=1.07; T-test=-4.637, df=98, p<0.001). The exclusion of the supernumerary chromosome from the analysis resulted in no statistically significant difference in the number of active NORs in autosomes in cells with (mean 4.194, SD=1.348) and without the B chromosome (T-test=0.545; df=98; p=0.587).

The banding pattern with DAPI/CMA_3_ was similar in all specimens and varied among chromosomes (Fig. 2g–j). The pericentromeric regions of different autosomes had a heterogeneous pattern of DAPI/CMA_3_ staining, which were negative, positive or neutral depending of the considered pair (Fig. 2g, i). In sex chromosomes, the pericentromeric regions of Xa and Xs were neutral with both fluorochromes, while the short arm of the Xs was DAPI negative/CMA_3_ positive (Fig. 2g–h). The Y chromosome showed a small interstitial DAPI positive band, being telomeres CMA_3_ positive and the centromere CMA_3_ neutral (Fig. 2h, j). The B chromosome showed a DAPI positive/CMA_3_ neutral band in the pericentromeric region (Fig. 4d–e).

**Figure 5.**
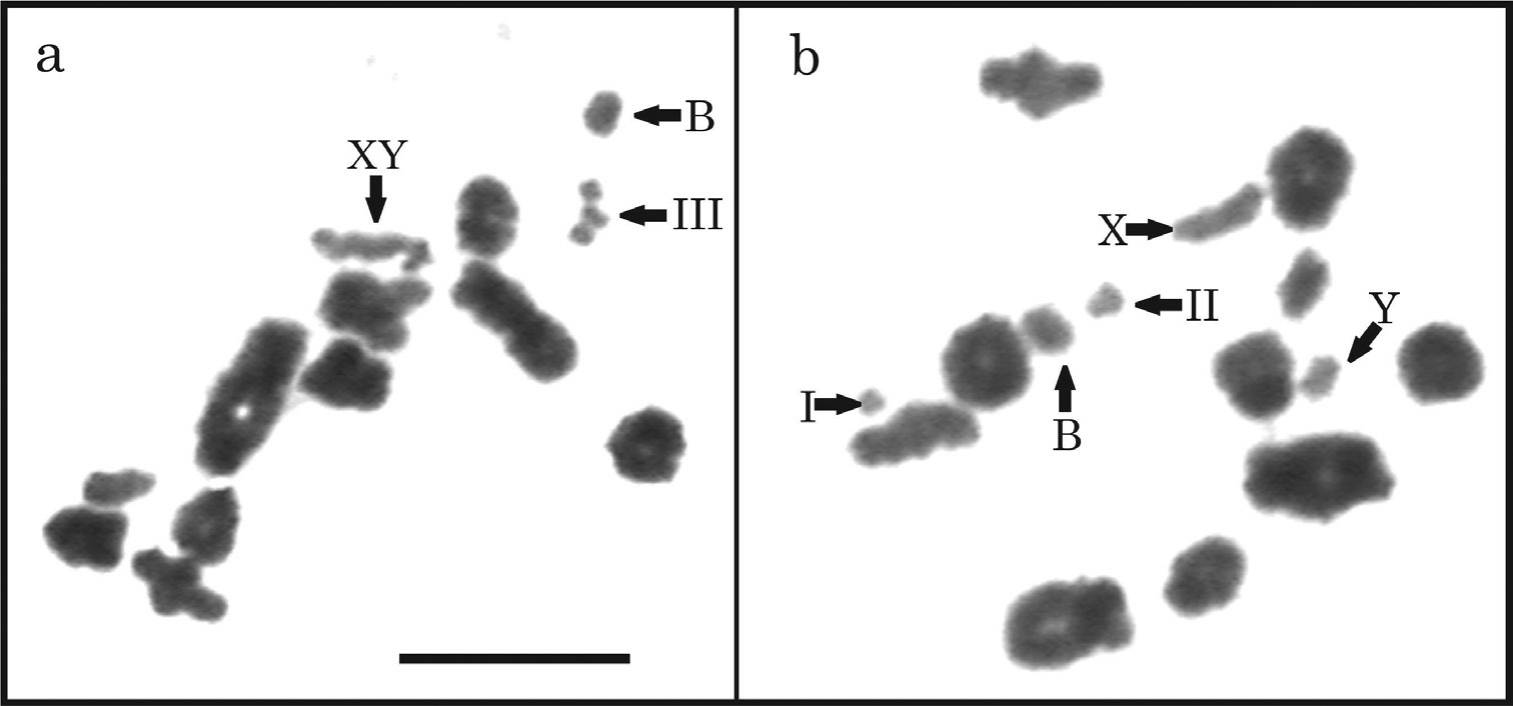
Diakinesis cells of an individual with trisomy and one B chromosome: **a** 10 autosomal bivalents, plus a trivalent (III) of chromosome 11, the sex pair XY and the supernumerary chromosome as univalent (B) **b** Note the presence of pair 11 as one bivalent (II) plus a univalent (I) and X chromosome dissociated of Y. Bar = 10 µm.

Meiotic cells of a male with 2n=24 showed 11 autosomal bivalents during diakinesis and one sex bivalent, which was recognized by its differential pyknosis, size and shape. From 30 studied cells in the specimen with trisomy with a B chromosome (2n=26), the three chromosomes 11 were observed either a trivalent (14/30) or as one bivalent plus a univalent (16/30) (Fig. 5). In addition, we observed a cell in which the X and Y chromosome were dissociated (Fig. 5b).

## Discussion

The studied populations of *Akodon montensis* from Brazil showed high chromosome variability (Kasahara and Yonenaga-Yassuda 1982; Fagundes et al. 2000). However in Argentina, with a low sample size, no karyotype variation had been detected previously (Liascovich and Reig 1989). In this work we found the same variability described in Brazil, which involve the presence of a B chromosome, X chromosomes variants and seeming XY females.


 Constitutive heterochromatin (CH) is in mammals, and particularly in rodents, an important source of karyotype variability (Graphodatsky et al. 2011). *Akodon montensis* has small positive C-bands in the pericentromeric regions of all chromosomes (Kasahara and Yonenaga-Yassuda 1982; this work), which is common in *Akodon* species, and in rodents in general (Ortiz et al. 1998; Lisanti et al. 2001; Ventura et al. 2006; Lanzone et al. 2011; Labaroni et al. 2014).

Patterns of fluorescent bands DAPI/CMA_3_ are comparable to G- and R-banding respectively (Veyrunes et al. 2007). Our results of DAPI staining showed high homology among karyotypes of specimens from Argentina and those for Brazil studied with G-banding method (Fagundes and Yonenaga-Yassuda 1998; Silva and Yonenaga-Yassuda 2004), which indicates a high conservation in the standard karyotype of this abundant and widely distributed species.

The XX/XY sex chromosome system is the most common among mammals, being males heterogametic and females homogametic. However, certain species depart from this pattern (Graphodatsky et al. 2011). In our sample two females presented heteromorphic sex chromosomes (XY). In *Akodon montensis* from Brazil the occurrence of XY female was confirmed with molecular cytogenetic techniques (Fagundes et al. 2000). In *Akodon*, some species contain a large proportion of XY fertile females (Hoekstra and Edwards 2000; Bianchi 2002). Even though, in *Akodon montensis* this condition has a relative low frequency (Fagundes et al. 2000; this work).

In Brazil and Argentina, two morphologies for the X chromosome were observed: acrocentric and subtelocentric (Kasahara and Yonenaga-Yassuda 1982; Fagundes et al. 2000; Di-Nizo et al. 2014; this work). This polymorphism has three possible combinations in females: homozygous acrocentric (XaXa) and subtelocentric (XsXs), and heterozygous (XaXs). The XsXs found in one female is reported for the first time. Females with XaXa were the most frequent in specimens studied here (56.25%) and in Brazil (75%) (Kasahara and Yonenaga-Yassuda 1982). Additionally, XY females with different types of X chromosomes were detected in both countries (Fagundes et al. 2000; this work). In males, we observed only the Xa; but in Brazilian populations males with both X types were found (Kasahara and Yonenaga-Yossuda 1982). Thus, the data suggest differences in the frequencies of X chromosome variants among populations, but larger sample sizes are needed to validate these observations.

Sex chromosomes of several rodents showed variation in the amount and distribution of heterochromatin (Patton and Sherwood 1983). In this work both Xa and Xs presented CH in the pericentromeric regions. Additionally, the short arms of Xs had positive C-bands. However, the data from different localities of Brazil are controversial. Some authors detected the same pattern described here (Fagundes et al. 2000); but in another study the short arm of Xs did not show CH (Kasahara and Yonenaga-Yassuda 1982). The Y chromosome of *Akodon montensis* from Argentina was completely heterochromatic. The same pattern was observed in several mammals, and particularly in individuals of *Akodon montensis* from Brazil (Kasahara and Yonenaga-Yassuda 1982; Waters et al. 2007). Although, Fagundes et al. (2000) described a non heterochromatic Y chromosome for *Akodon montensis*.

B chromosomes (Bs) appear as supernumerary elements to the standard chromosome complement and are highly variables (Silva and Yonenaga 2004; Vujošević and Blagojević 2004; Ventura et al. 2015). The B of *Akodon montensis* studied here had identical morphology to those detected in Brazil (Yonenaga-Yassuda et al. 1975; Kasahara and Yonenaga-Yassuda 1982; Yonenaga-Yassuda et al. 1992; Fagundes et al. 2000; Silva and Yonenaga-Yassuda 2004). However, the described C- and G-banding patterns varied in different studies. Some authors described the B chromosome as slightly heterochromatic and uniformly G-banded (Kasahara and Yonenaga-Yassuda 1982; Silva and Yonenaga-Yassuda 2004); while others reported it as almost heterochromatic with conspicuous pericentromeric C-bands (Kasahara 2009). The B studied here had two heterochromatic bands (pericentromeric and interstitial), which were partially DAPI positive/CMA_3_ neutral. CH patterns on Bs have been extensively studied in some species of rodents, in which most often appear as almost completely heterochromatic. Additionally, in some cases Bs vary within and among populations, as in *Perognathus baileyi* (Merriam, 1894) and *Nectomys squamipes* Brants, 1827 (Silva and Yonenaga-Yassuda 2004; Vujošević and Blagojević 2004). In *Akodon montensis*, the described patterns suggest that different polymorphisms for B chromosomes may be coexisting in this species.

In *Akodon montensis* the B chromosome showed NORs at the end of both arms, which are also coincident with the location of rDNA detected by fluorescent *in situ* hybridization (Kasahara 2009). The presence of Ag-NORs in Bs has been described in other rodent species such as *Sooretamys angouya* (Fischer, 1814) and *Apodemus peninsulae* (Thomas, 1907) (Silva and Yonenaga-Yassuda 2004; Vujošević and Blagojević 2004). In *Akodon montensis* from Brazil was detected a low frequency of Bs with NOR activity, where only one of four analyzed individuals presented Ag-NOR marks (Yonenaga Yassuda et al. 1992). In this work B-chromosome had Ag-NOR marks in one or both ends in high frequency, which lead to a higher average of active NORs in the cells. These observations support the hypothesis that different B chromosomes can be present in *Akodon montensis*.

Variation in the frequency of B chromosomes is common among populations (Silva and Yonenaga Yassuda 2004; Vujošević and Blagojević 2004; Ventura et al. 2015). In *Akodon montensis* the frequency of individuals with Bs appears to vary among localities, but several populations were studied with low sample size. In this study the total frequency of individuals with a B chromosome was 19%. Compiled data from Brazil (N=346) calculated a total frequency of 28.13% for individuals with 1 B, 2.27% with two Bs, and 0.28% with unstable Bs that formed a mosaic of 1B-2Bs (Silva and Yonenaga-Yassuda 2004). In this work individuals with more than one B were not identified. This chromosome was stable in mitoses and meioses, since no evidence for accumulation or elimination were detected.

Finally, in the present paper we report for the first time a trisomy of chromosome 11 in a single individual. In *Akodon cursor* also were observed an individual with trisomy for chromosome 7 (Fagundes et al. 1998). No phenotypic malformations were detected in both cases. However the frequency of trisomies in natural populations and the biological consequences of this condition have not been investigated yet.

In conclusion, chromosome data for *Akodon montensis* showed high variability in all studied populations throughout its geographic range. However, additional data are needed to understand the dynamic of the multiple chromosome polymorphism observed in this species of sigmodontine rodents.

## Supplementary materials

Supplementary material 1 Pattern of Ag-NOR´s distribution in *Akodon montensis* from Argentina analyzed in this work. Data type: Chromosome pairs with positive signals after silver staining. Explanation note: 1 HOM = only one homologue was marked, 2 HOM = both homologues were marked. 2 Telo = both ends of the B chromosome were marked; 1 Telo = only one end of the B chromosome was marked, being “p” when the short arm was marked and “q” when the long arm was marked. Individuals from 1 to 10 had no supernumerary chromosome and from 11 to 14 had the B chromosome. Total = total number of Ag-NOR marks in each cell; Total without B = total number of Ag-NOR marks excluding that of the B chromosome. This dataset is made available under the Open Database License (http://opendatacommons.org/licenses/odbl/1.0/). The Open Database License (ODbL) is a license agreement intended to allow users to freely share, modify, and use this Dataset while maintaining this same freedom for others, provided that the original source and author(s) are credited.Matías Maximiliano Malleret, Carolina Alicia Labaroni, Gabriela Verónica García, Juan Martín Ferro, Dardo Andrea Martí, Cecilia Lanzone
