# Supplementary material for: Chromosomal variation in Argentine populations of Akodon montensis Thomas, 1913 (Rodentia, Cricetidae, Sigmodontinae)
Source: Comp Cytogenet. 2016 Feb 2;10(1):129–40. doi: 10.3897/CompCytogen.v10i1.6420 (PMC4856931; doi:10.3897/CompCytogen.v10i1.6420)
Supplement: Supplementary material 1 — Pattern of Ag-NOR´s distribution in Akodon montensis from Argentina analyzed in this work. [file CompCytogen-010-129-s001.pdf]

Individual	Cell	Pair 2	Pair 4	Pair 6	Pair 7	Pair 10	B	Total	Total without B
1	1	-	1 HOM	1 HOM	-	2 HOM	-	4	
1	2	1 HOM	1 HOM	1 HOM	-	1 HOM	-	4	
1	3	1 HOM	1 HOM	1 HOM	-	2 HOM	-	5	
1	4	1 HOM	1 HOM	1 HOM	-	2 HOM	-	5	
1	5	1 HOM	1 HOM	-	-	2 HOM	-	4	
1	6	1 HOM	1 HOM	1 HOM	-	2 HOM	-	5	
2	1	-	1 HOM	-	-	1 HOM	-	2	
2	2	-	1 HOM	-	1 HOM	1 HOM	-	3	
2	3	-	1 HOM	1 HOM	1 HOM	-	-	3	
2	4	1 HOM	2 HOM	-	-	1 HOM	-	4	
2	5	-	2 HOM	1 HOM	-	1 HOM	-	4	
2	6	1 HOM	1 HOM	1 HOM	-	2 HOM	-	5	
2	7	-	1 HOM	1 HOM	1 HOM	1 HOM	-	4	
2	8	1 HOM	1 HOM	2 HOM	-	1 HOM	-	5	
3	1	2 HOM	-	-	1 HOM	1 HOM	-	4	
3	2	2 HOM	-	1 HOM	-	1 HOM	-	4	
3	3	2 HOM	-	2 HOM	-	2 HOM	-	6	
3	4	1 HOM	2 HOM	1 HOM	-	1 HOM	-	5	
3	5	1 HOM	1 HOM	1 HOM	-	1 HOM	-	4	
4	1	-	1 HOM	2 HOM	-	2 HOM	-	5	
4	2	1 HOM	2 HOM	1 HOM	-	2 HOM	-	6	
4	3	-	-	1 HOM	-	1 HOM	-	2	
4	4	1 HOM	1 HOM	1 HOM	-	1 HOM	-	4	
4	5	-	1 HOM	1 HOM	-	2 HOM	-	4	
5	1	1 HOM	-	-	-	2 HOM	-	3	
5	2	1 HOM	1 HOM	1 HOM	-	1 HOM	-	4	
5	3	2 HOM	-	2 HOM	-	1 HOM	-	5	
5	4	1 HOM	-	-	-	2 HOM	-	3	
5	5	1 HOM	1 HOM	1 HOM	-	1 HOM	-	4	
6	1	1 HOM	2 HOM	-	-	1 HOM	-	4	
6	2	1 HOM	1 HOM	1 HOM	-	1 HOM	-	4	
6	3	1 HOM	1 HOM	1 HOM	-	2 HOM	-	5	
6	4	1 HOM	1 HOM	1 HOM	-	2 HOM	-	5	
6	5	1 HOM	2 HOM	-	-	1 HOM	-	4	
6	6	1 HOM	2 HOM	1 HOM	-	2 HOM	-	6	
6	7	1 HOM	1 HOM	2 HOM	1 HOM	1 HOM	-	6	
6	8	2 HOM	-	1 HOM	1 HOM	2 HOM	-	6	
7	1	2 HOM	1 HOM	1 HOM	-	2 HOM	-	6	
7	2	-	1 HOM	1 HOM	-	2 HOM	-	4	
7	3	-	1 HOM	2 HOM	-	1 HOM	-	4	

7	4	1 HOM	2 HOM	2 HOM	-	1 HOM	-	6	
7	5	-	2 HOM	1 HOM	-	2 HOM	-	5	
8	1	1 HOM	-	1 HOM	-	1 HOM	-	3	
8	2	1 HOM	-	1 HOM	1 HOM	1 HOM	-	4	
8	3	1 HOM	2 HOM	1 HOM	-	2 HOM	-	6	
8	4	-	1 HOM	1 HOM	-	1 HOM	-	3	
8	5	1 HOM	-	2 HOM	-	1 HOM	-	4	
9	1	1 HOM	1 HOM	-	-	2 HOM	-	4	
9	2	-	1 HOM	1 HOM	-	2 HOM	-	4	
9	3	1 HOM	1 HOM	-	-	1 HOM	-	3	
9	4	-	1 HOM	-	1 HOM	2 HOM	-	4	
9	5	-	1 HOM	-	-	2 HOM	-	3	
9	6	1 HOM	1 HOM	-	-	1 HOM	-	3	
9	7	1 HOM	1 HOM	-	-	2 HOM	-	4	
9	8	1 HOM	1 HOM	-	-	2 HOM	-	4	
9	9	2 HOM	2 HOM	1 HOM	-	1 HOM	-	6	
9	10	1 HOM	2 HOM	-	-	1 HOM	-	4	
10	1	1 HOM	1 HOM	-	-	1 HOM	-	3	
10	2	1 HOM	1 HOM	2 HOM	-	2 HOM	-	6	
10	3	1 HOM	1 HOM	2 HOM	-	2 HOM	-	6	
10	4	1 HOM	2 HOM	1 HOM	-	1 HOM	-	5	
10	5	1 HOM	1 HOM	-	-	1 HOM	-	3	
10	6	1 HOM	2 HOM	2 HOM	-	1 HOM	-	6	
10	7	1 HOM	2 HOM	-	-	1 HOM	-	4	
11	1	1 HOM	2 HOM	-	-	1 HOM	2 TELO	6	4
11	2	-	2 HOM	1 HOM	-	2 HOM	1 TELO "q"	6	5
11	3	-	1 HOM	1 HOM	-	-	2 TELO	4	2
11	4	1 HOM	1 HOM	-	-	2 HOM	2 TELO	6	4
11	5	2 HOM	-	-	1 HOM	2 HOM	2 TELO	7	5
11	6	-	1 HOM	-	-	1 HOM	-	2	2
11	7	-	2 HOM	-	-	1 HOM	2 TELO	5	3
11	8	-	1 HOM	-	-	1 HOM	-	2	2
11	9	-	1 HOM	-	-	1 HOM	-	2	2
11	10	-	2 HOM	2 HOM	-	1 HOM	2 TELO	7	5
12	1	1 HOM	1 HOM	1 HOM	-	1 HOM	-	4	4
12	2	1 HOM	2 HOM	1 HOM	-	-	-	4	4
12	3	1 HOM	2 HOM	-	-	-	2 TELO	5	3
12	4	1 HOM	1 HOM	-	-	1 HOM	2 TELO	5	3
12	5	2 HOM	1 HOM	1 HOM	-	-	-	4	4
12	6	2 HOM	1 HOM	2 HOM	-	-	-	5	5
12	7	2 HOM	2 HOM	1 HOM	-	-	-	5	5
12	8	2 HOM	2 HOM	-	-	-	2 TELO	6	4
12	9	1 HOM	2 HOM	-	-	1 HOM	1 TELO "p"	5	4

13	1	-	1 HOM	1 HOM	1 HOM	2 HOM	2 TELO	7	5
13	2	-	1 HOM	2 HOM	-	2 HOM	2 TELO	7	5
13	3	-	1 HOM	2 HOM	-	1 HOM	1 TELO "q"	5	4
13	4	-	1 HOM	-	-	1 HOM	2 TELO	4	2
13	5	-	1 HOM	-	-	1 HOM	2 TELO	4	2
13	6	-	1 HOM	1 HOM	-	2 HOM	2 TELO	6	4
13	7	-	1 HOM	2 HOM	-	2 HOM	2 TELO	7	5
14	1	1 HOM	2 HOM	1 HOM	-	1 HOM	2 TELO	7	5
14	2	1 HOM	2 HOM	1 HOM	-	1 HOM	1 TELO "p"	6	5
14	3	-	2 HOM	1 HOM	-	1 HOM	2 TELO	6	4
14	4	1 HOM	1 HOM	2 HOM	-	1 HOM	2 TELO	7	5
14	5	1 HOM	2 HOM	2 HOM	-	2 HOM	2 TELO	9	7
14	6	-	1 HOM	1 HOM	1 HOM	1 HOM	2 TELO	6	4
14	7	1 HOM	2 HOM	2 HOM	-	1 HOM	2 TELO	8	6
14	8	1 HOM	2 HOM	1 HOM	-	2 HOM	2 TELO	8	6
14	9	1 HOM	2 HOM	2 HOM	-	2 HOM	2 TELO	9	7
14	10	1 HOM	2 HOM	1 HOM	-	1 HOM	2 TELO	7	5

Supplementary files 1: Pattern of Ag-NOR´s distribution in *Akodon montensis* from Argentina analyzed in this work. 1 HOM= only one homologue was marked, 2 HOM= both homologues were marked. 2 Telo= both ends of the B chromosome were marked; 1 Telo= only one end of the B chromosome was marked, being “p" when the short arm was marked and “q" when the long arm was marked. Individuals from 1 to 10 had no supernumerary chromosome and from 11 to 14 had the B chromosome. Total= total number of Ag-NOR marks in each cell; Total without B=total number of Ag-NOR marks excluding that of the B chromosome.
